# A comparative review of time-resolved x-ray and electron scattering to probe structural dynamics

**DOI:** 10.1063/4.0000249

**Published:** 2024-05-01

**Authors:** Yunbeom Lee, Key Young Oang, Doyeong Kim, Hyotcherl Ihee

**Affiliations:** 1 Center for Advanced Reaction Dynamics (CARD), Institute for Basic Science (IBS), Daejeon 34141, South Korea; 2 Department of Chemistry, Korea Advanced Institute of Science and Technology (KAIST), Daejeon 34141, South Korea; 3 Radiation Center for Ultrafast Science, Korea Atomic Energy Research Institute (KAERI), Daejeon 34057, South Korea

## Abstract

The structure of molecules, particularly the dynamic changes in structure, plays an essential role in understanding physical and chemical phenomena. Time-resolved (TR) scattering techniques serve as crucial experimental tools for studying structural dynamics, offering direct sensitivity to molecular structures through scattering signals. Over the past decade, the advent of x-ray free-electron lasers (XFELs) and mega-electron-volt ultrafast electron diffraction (MeV-UED) facilities has ushered TR scattering experiments into a new era, garnering significant attention. In this review, we delve into the basic principles of TR scattering experiments, especially focusing on those that employ x-rays and electrons. We highlight the variations in experimental conditions when employing x-rays vs electrons and discuss their complementarity. Additionally, cutting-edge XFELs and MeV-UED facilities for TR x-ray and electron scattering experiments and the experiments performed at those facilities are reviewed. As new facilities are constructed and existing ones undergo upgrades, the landscape for TR x-ray and electron scattering experiments is poised for further expansion. Through this review, we aim to facilitate the effective utilization of these emerging opportunities, assisting researchers in delving deeper into the intricate dynamics of molecular structures.

## I. INTRODUCTION

The structure of a molecule is one of the key parameters that determine the properties of the molecule, such as charge distribution, dipole moment, and chemical reactivity.^1,2^ In particular, the structures of reaction intermediates influence the reaction pathway, thereby affecting the reaction products and their yields. For this reason, elucidating the structures of molecules and the dynamic behavior of their structures is essential for understanding chemical reactions. Traditionally, time-resolved (TR) spectroscopy using the pump–probe approach has been employed to study the dynamic behavior of molecules.^3–7^ Still, spectroscopic signals primarily provide information associated with the energy levels of molecules, making it challenging to obtain direct information about the changes in the molecular structures. In contrast, scattering signals, generated by particles such as photons and electrons scattered by molecules, directly relate to the molecular structure rather than energy levels. Therefore, TR scattering can provide direct information about the changes in molecular structures. In this sense, TR scattering and TR spectroscopy offer complementary information, with TR scattering being particularly valuable for directly probing changes in molecular structures.

To investigate the detailed structures of molecules using scattering signals, the wavelengths of the scattering particles should be comparable to or smaller than interatomic distances. Particles, such as x-ray photons, electrons, and neutrons, meet this criterion and are commonly employed as scattering particles for probing molecular structures. For their application in TR scattering experiments, these particles must meet an additional requirement. In TR scattering experiments, it is essential to collect scattering signals at multiple time delays, a task that demands more time compared to static scattering experiments. Considering this, TR scattering experiments are typically performed under conditions conducive to acquiring data with a high signal-to-noise ratio within a specified timeframe. To rapidly improve the signal-to-noise ratio, the scattering particles should exhibit strong scattering powers or be generated in a substantial number within a unit time. X-ray and electron sources satisfy these conditions, rendering them suitable for TR scattering experiments. Furthermore, the advancement of facilities for generating x-rays and electrons has accelerated the application of these particles in TR scattering experiments. For example, advancements in the development of plasma x-ray sources since the 1990s have enabled the utilization of x-ray pulses with sub-picosecond pulse widths for TR x-ray scattering experiments.^8–11^ Other notable examples include the development of Sub-Picosecond Pulse Sources (SPPS),^12–14^ followed by the rise of x-ray free-electron lasers (XFELs) since the 2000s.^15–19^ Such advancement has significantly enhanced the temporal resolutions of TR x-ray scattering experiments to the level of 100 femtoseconds (fs) or superior. This groundbreaking achievement has paved the way for in-depth investigations into ultrafast dynamics, encompassing phenomena such as phase transition, changes in chemical bonds, and the dynamics of wavepackets such as vibrations. Similarly, ultrafast electron diffraction (UED) has significantly advanced with the establishment of mega-electron-volt UED (MeV-UED) facilities using a radio frequency (RF) electron gun, which can generate exceptionally bright and temporally short electron pulses compared to traditional kilo-electron-volt UED (keV-UED) setups employing a direct current (DC) electron gun.^20^

While both x-rays and electrons can be utilized in TR scattering experiments, offering valuable structural information, they also present distinct features. The most distinct differences between electrons and x-rays lie in their scattering powers and charges. These dissimilarities necessitate distinct experimental conditions for TR scattering experiments using x-rays and electrons, influencing factors such as sample thickness, the overall experimental environment, beam focusing, and pulse duration. In Sec. IV, we will delve into these differences in detail. Simultaneously, in this review, we briefly touch on the concept of x-ray and electron scattering, several methods used for the analysis, and state-of-the-art facilities for TR x-ray and electron scattering as well. In addition, TR x-ray and electron scattering experiments conducted at those facilities will also be reviewed with emphasis on the sample phases and the observed dynamics. With this, we aim to guide prospective researchers in selecting the most suitable experiment tailored to their specific objectives.

## II. SCATTERING OF X-RAY AND ELECTRON

Scattering is the phenomenon where the trajectory of a particle is altered due to its interaction with other objects. Scattering can be categorized into elastic scattering, where the energy of the scattering particle remains unchanged before and after scattering, and inelastic scattering, where the energy changes. In studies focusing on molecular structures, it is typical to utilize elastic scattering signals. Accordingly, this review will focus specifically on elastic scattering. A similar term for scattering is diffraction. Although the distinction between scattering and diffraction is not always clear, phenomena caused by amorphous objects and fluids such as liquids are generally referred to as scattering, while those caused by objects with a periodic arrangement, such as crystals, are termed diffraction. Here, we will use scattering as a representative terminology for the phenomenon occurring with various objects, reserving the use of diffraction exclusively for cases involving crystals. Nevertheless, in the realm of electron scattering, the term “diffraction” is frequently used to describe the scattering from non-crystal samples such as gases. This follows a common convention, wherein the term “ultrafast electron diffraction” is used to refer to ultrafast TR electron scattering, regardless of the phases of the samples. The rationale behind the convention of referring to electron scattering from a non-crystal sample as diffraction is not clear. We speculate that this convention arises due to the characteristics of electron scattering signals. In comparison to x-rays, the form factor for electrons, whose square can represent the scattering signal, undergoes more rapid changes with variations in the scattering angle. Consequently, the interference pattern in the scattering signal, arising from the atomic pairs in a molecule, also exhibits a sharper rise and decay for electrons compared to x-rays. In other words, the interference pattern in the scattering signal is clearer and sharper for electrons. Such a sharp interference pattern is similar to the characteristics observed in Bragg spots due to diffraction. The rapid change of the form factor and sharp interference pattern of electron scattering intensity will be further discussed in Sec. III A 2.

The scattering signals of the x-rays and electrons are in reciprocal relation to the molecular structures. Specifically, for x-rays, the form factor of an atom in reciprocal space (commonly referred to as *q* space) is in reciprocal relation to the electron density in real space.^21–26^ Meanwhile, in the case of electrons, the form factor of an atom is reciprocally related to the electrostatic potential in real space.^25–27^ Likewise, the structure factor of a molecule, which can be regarded as a form factor of a multi-atom system, has a reciprocal relation to the electron density (for x-ray) or electrostatic potential (for electrons) of a molecule. The structure factor, derived from the scattering signal of a sample, can be employed to determine the electron density, electrostatic potential, and ultimately the molecular structures. In this manner, information about the molecular structure in real space can be deduced from the scattering signal.

### A. Time-resolved x-ray and electron scattering

**FIG. 1.**
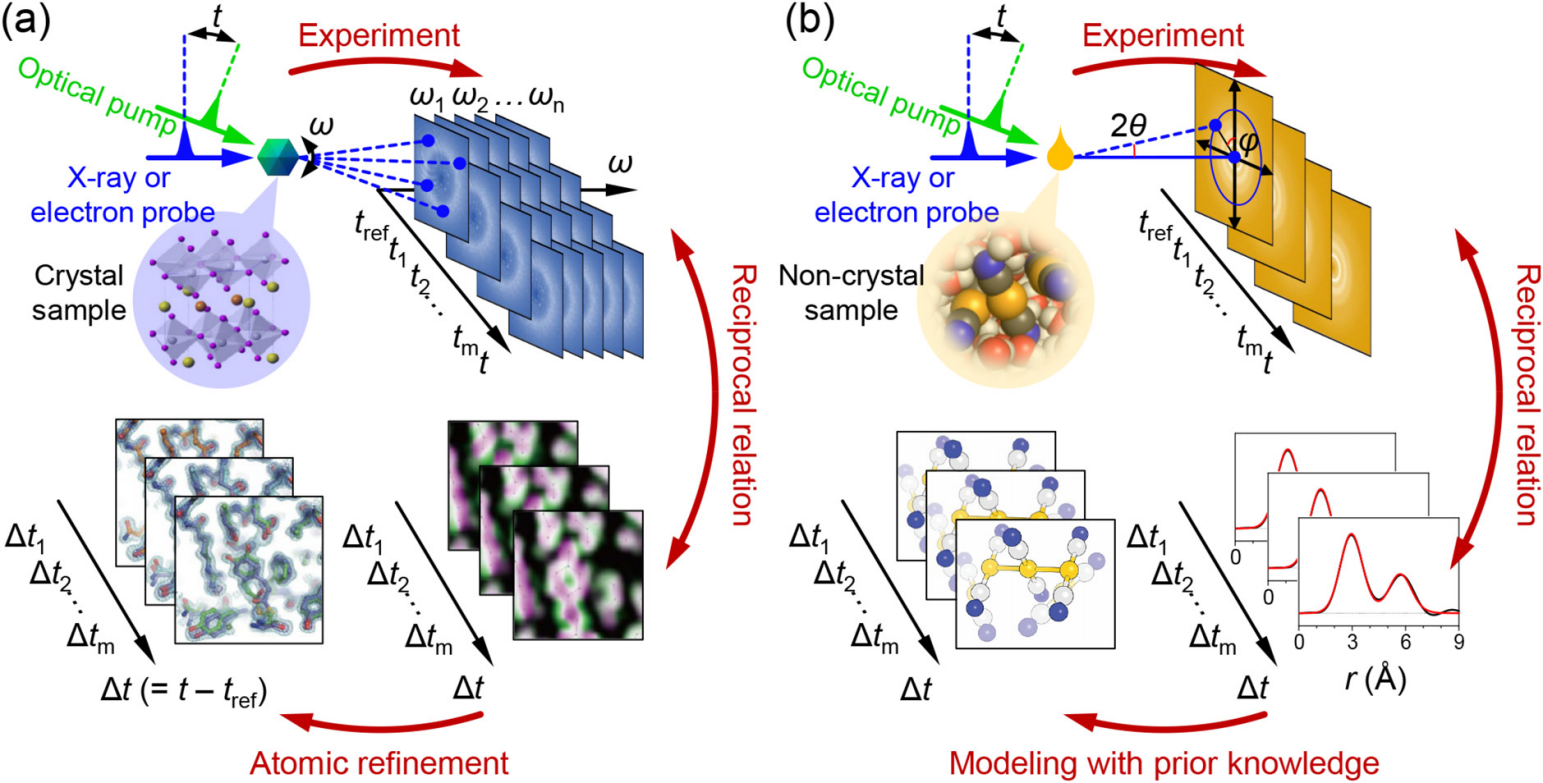
Illustration of the typical experimental setup for time-resolved (TR) x-ray and electron scattering on (a) a single crystal using crystallography and (b) non-crystal samples such as liquids and gases. In a TR x-ray and electron scattering experiment, the reaction of the sample is triggered by the pump, which is typically an optical laser pulse. Subsequently, the scattering signal of the probe (x-ray or electron) from the sample is tracked over time using a 2D area detector. The scattering signals are in reciprocal relation to the electron density (for x-rays) and electrostatic potential (for electrons). In crystallography, collecting scattering signals at various crystal orientations (*ω*) enables the extraction of 3D information on electron density or electrostatic potential, leading to a 3D molecular structure through structure refinement. In contrast, scattering signals from other non-crystal phases offer 1D information in real space. Therefore, it is customary to identify a model structure that best describes the experimental scattering signal based on prior knowledge, rather than directly obtaining the 3D molecular structure from the scattering signal. For samples with randomly oriented molecules such as liquids and gases, scattering depends solely on the scattering angle (2*θ*). However, in cases where molecules are not randomly oriented, the scattering signal exhibits dependence on another angle (*φ*) related to the detector, in addition to 2*θ*. As only a small fraction of the molecules in the sample undergo the reaction, the change in the scattering signal over time is typically small. To clarify the minute change in the scattering signal, it is conventional to generate and analyze the difference scattering signal, representing the difference between the scattering signals at time *t* and the reference time (*t*_ref_).

TR x-ray and electron scattering experiments are methods that combine the pump–probe scheme and scattering. In these experiments, the reaction of the sample is initiated with the pump, and then the changes in the scattering signal of the probe due to the sample are measured over time. Since the scattering signal of the probe contains information about the molecular structure in the sample, analyzing it allows the observation of how the molecular structure changes with the progress of the reaction. An optical laser pulse is commonly used as the pump, while a pulse of x-rays or electrons is frequently used as the probe. A typical scheme for a TR x-ray and electron scattering experiment is illustrated in Fig. 1.

As scattering can be applied to samples in various phases to determine molecular structures, the application of TR x-ray and electron scattering is also versatile across samples with different phases. Still, the detailed experimental scheme and analysis methods may vary depending on the phase of the sample. Among the phases of the samples, single crystals exhibit a distinct advantage in elucidating molecular structures. Diffraction signals from a single crystal can be used to obtain information about a complete 3D molecular structure, provided that the diffraction signals covering a sufficient fraction of reciprocal space are collected.^22–24,28^ To achieve this, diffraction signals are typically collected at various orientations of a crystal to encompass a comprehensive reciprocal space. The method of obtaining a 3D molecular structure by collecting diffraction signals in this manner is referred to as crystallography. Leveraging the advantage of diffraction signals from single crystals, TR x-ray crystallography has been successfully applied to diverse protein and small molecule samples, allowing for the visualization of changes in complete 3D molecular structures over time.^29–31^ Alternatively, it is possible to deduce the structural changes of a single crystal even with a small number of Bragg spots, although the structural information is somewhat limited compared to crystallography. A thin film of a single crystal, a primary focus in TR electron diffraction experiments, serves as a representative example for the application of this approach.^32–51^ In typical TR electron diffraction experiments on thin films of single crystals, researchers commonly track intensity changes in a few Bragg spots to investigate structural changes in the thin films. Studies reporting the direct acquisition of 3D molecular structures of the thin films are currently limited due to the often insufficient information in the diffraction signals obtained from thin films of single crystals. Nevertheless, in principle, it is possible to acquire 3D molecular structures even for thin films of single crystals as long as diffraction signals from a sufficient portion of reciprocal space are obtained.

For polycrystalline, liquid, and gas samples, unlike in the case of single crystals, the random or less ordered orientation of molecules in the samples leads to obtaining only a 1D pair distribution function, which indicates interatomic distances, from the scattering signal.^28^ In such cases, to obtain the real-space molecular structure from the scattering signal, it is often necessary to establish a model for the molecular structure. Subsequently, one needs to calculate the theoretical scattering intensity from this model structure. The final structure is derived by either (1) selecting a model structure, among the available options, whose theoretical scattering intensity best describes the experimental data or (2) refining the model structure to ensure its theoretical scattering intensity exhibits satisfactory agreement with the experimental scattering signal.

In Sec. III, we will briefly present representative analysis methods for TR x-ray and electron scattering. As mentioned earlier, the analysis methods of TR scattering data depend on the phases of samples. Our discussion in Sec. III will be limited to relatively simple systems, namely, gases and liquid solutions. For such systems, two commonly employed methods for calculating scattering signals from the model structure are using (i) the Debye equation based on independent atomic model (IAM) and (ii) *ab initio* calculations. Each of these methods will be discussed in Sec. III.

### B. Measuring the scattering signal

Before discussing the approaches for analysis of the scattering signal, it would be beneficial to elaborate on how the experimental scattering signal is obtained. The scattering signal can be measured through several methods, but a commonly employed approach for measuring TR x-ray and electron scattering signals involves the use of a 2D area detector (Fig. 1). Raw scattering signals collected on a 2D area detector are influenced by various factors unrelated to the characteristics of the scattering sample, such as the x-ray polarization (in the case of x-rays) and scattering angle covered by a unit area of the detector. Typically, correction processes are applied to the scattering signals to eliminate these effects, and several programs are available for such a purpose.^52,53^

Scattering signals from samples with randomly oriented molecules usually appear isotropic on a 2D area detector. In other words, the scattering intensity depends solely on the scattering angle, and all positions on the detector with the same scattering angle show the same scattering intensity. However, for samples with nonrandomly oriented molecules, the scattering signal may exhibit anisotropy on a 2D area detector.^54–56^ This implies that different detector positions may yield different scattering intensities even when sharing the same scattering angle. Frequently, the orientations of molecules, whose reactions are initiated by a linearly polarized optical laser pulse in a TR scattering experiment, are temporarily not random. Therefore, in TR scattering studies, especially those conducted at XFELs or MeV-UED facilities focusing on the ultrafast dynamics, anisotropic scattering signals are often observed.^57–69^ In these cases, it is common to decompose the total scattering signal into isotropic and anisotropic scattering components for analysis. Details about the decomposition of the scattering signals will be discussed in Sec. III A 3.

## III. METHODS TO CALCULATE SCATTERING INTENSITIES

### A. Independent atomic model

IAM assumes that each atom in a molecule is spherical and independent. The term “spherical” indicates that the electron density (for x-ray scattering) and electrostatic potential (for electron scattering) are isotropic in space. The term “independent” means that the atoms in a molecule do not influence each other, implying that the electron density due to the bonding character between atoms is neglected. For example, when considering methane and methanol, IAM treats them as having the same carbon atom, regardless of the distinct connectivities of the carbon atoms in two molecules. In other words, it assumes that each carbon atom in the molecules does not interact with its surroundings. A widely adopted method for describing scattering intensity based on IAM is employing the Debye equation. IAM is also employed in crystallography to obtain a primary crystal structure, while more advanced methods can be used to accurately depict the aspherical nature of the electron density distributions of atoms in a molecule.

#### 1. Debye equation

The Debye equation is a formula used to calculate the scattering intensity of an ensemble of molecules with random orientations. Namely, the scattering intensity obtained from the Debye equation represents the isotropic scattering intensity. In the Debye equation, the scattering intensity is described as a function of the magnitude of the momentum transfer vector (*q*), which is defined as follows.

$$q=\frac{4\pi \;\text{sin}(\frac{2\theta }{2})}{\lambda }.$$ (1)

Here, 2*θ* is the scattering angle [Fig. 1(b)] and *λ* is the wavelength of the scattering particle. According to the Debye equation, the scattering intensity [*I*(*q*)] relies on the form factor of each atom (*f*) and the interatomic distances (*r_ij_*) as follows:

$$I(q)=I_{A}+I_{M}=\sum_{i}f_{i}{\left(q\right)}^{2}+\sum_{i}\sum_{j\neq i}f_{i}(q)f_{j}(q)\frac{\;\text{sin}(qr_{ij})}{qr_{ij}}.$$ (2)

Here, *I_A_* indicates the contribution of individual atoms (atomic contribution) to the scattering intensity, and *I_M_* indicates the contribution of atomic pairs within a molecule (molecular contribution). A form factor is related to the scattering power of an atom and is element-specific, meaning it remains the same regardless of the environment around the atom. In other words, the Debye equation considers a molecule as an assembly of independent atoms.

The equation described in Eq. (2) assumes a scenario where the distances between two atoms are fixed, meaning that *r_ij_*s in Eq. (2) have fixed values. In reality, the distances are not fixed at specific values but instead exhibit a distribution due to vibrational motions. If the effect of the distance distribution on the scattering intensity is not negligible, it is necessary to account for the distribution of the distances in the Debye equation. The modified Debye equation is expressed as follows:

$$I(q)=\sum_{i}f_{i}{\left(q\right)}^{2}+\sum_{i}\sum_{j\neq i}f_{i}(q)f_{j}(q)\frac{\;\text{sin}(qr_{ij})}{qr_{ij}}e^{-\frac{l_{ij}^{2}q^{2}}{2}}.$$ (3)

Here, $l_{ij}^{2}$ represents the mean squared displacement of *r_ij_*, and the term, $e^{-\frac{l_{ij}^{2}q^{2}}{2}}$, corresponds to the crystallographic Debye–Waller factor. The Debye–Waller factor in Eq. (3) is slightly different from the one used in crystallography. The crystallographic Debye–Waller factor employs the mean squared displacement of an atomic position rather than a bond length and is applied to each atomic form factor rather than a term related to the bond length.

As mentioned earlier, the Debye equation can be used to describe the scattering intensities of molecules with random orientations. Generally, in the ground state (without any perturbation), this condition is satisfied in samples such as liquids and gases. On the contrary, in the presence of perturbations such as the excitation by a linearly polarized light, random orientations may not be maintained. In the case of photoexcitation, the excitation probability varies with the angle between the transition dipole moment of a molecule and the polarization of the light. Consequently, in the excited state, molecules with specific orientations may be more prevalent than others. In such scenarios, the simple Debye equation becomes inapplicable, necessitating an alternative approach. Details regarding this matter will be explained in Sec. III A 3.

#### 2. Form factors for x-ray and electron scattering

##### a. Form factors for x-ray scattering

The form factor of an atom for x-ray scattering is commonly represented as a combination of Gaussian functions with respect to *q* and a constant. These parameters are typically fitted to the experimental form factor data or computed using the wavefunctions of corresponding atoms. The parameters (*a_i_*, *b_i_*, and *d*) to calculate the form factor are available in the literature.^70,71^

$$f^{X}(q)=\sum_{i}a_{i}e^{-b_{i}s^{2}}+d.$$ (4)

Here, *s* is *q* divided by 4*π*. It should be noted that the usage of *q* and *s* symbols may vary depending on the study. For instance, in certain studies, especially those related to UED, *s* is utilized to represent the magnitude of the momentum transfer vector, a term designated as *q* in this review. Consequently, readers should be attentive to the specific definition of each symbol in the context of the respective study. Although not explicitly evident in Eq. (4), one characteristic of the form factor in x-ray scattering is that it converges to the atomic number (*Z*) as *q* approaches zero. As mentioned earlier, the atomic form factor in x-ray scattering has a reciprocal relationship with the electron density of an atom. More specifically, the atomic form factor can be obtained by performing Fourier transform on the atomic electron density as follows:

$$f^{X}(q)=\int \rho (x)e^{-iq\cdot x}dV.$$ (5)

Here, *ρ* is the electron density of an atom, and **q** and **x** indicate the momentum transfer vector and positional vector, respectively. *V* represents the volume in real space. As seen in Eq. (5), when **q** equals 0 (where its magnitude, *q*, is also 0), the atomic form factor for x-ray is the integration of the atomic electron density over space, which equals *Z*.

The form factor for x-ray scattering is expressed relative to an electron, describing the scattering amplitude of an atom compared to an electron. To obtain an absolute value, the form factor should be multiplied by the classical radius of the electron (*r_e_*), which is expressed by the formula as follows:

$$r_{e}=\frac{1}{4\pi \varepsilon_{0}}\frac{e^{2}}{m_{e}c^{2}}.$$ (6)

Here, *ε*_0_ is the vacuum permittivity, *m_e_* is the rest mass of an electron, *e* is the elementary charge, and *c* is the speed of light. The value of *r_e_* is approximately 2.818 × 10^−15^ m. *r_e_* is also referred to as the Thomson scattering length and represents the scattering amplitude of a free electron.

##### b. Form factors for electron scattering

###### (1) Mott–Bethe formula

One of the most widely used methods for calculating the form factor for electron scattering is based on the Mott–Bethe formula. The Mott–Bethe formula is an approximation for calculating the form factor for electron scattering based on its x-ray counterpart. Unlike x-rays, electrons are charged particles that engage in Coulomb interactions not only with the electrons but also with the nucleus in an atom. Consequently, the computation of the form factor for electron scattering requires the consideration of the nuclear charge (*Z*), a crucial aspect embedded in the Mott–Bethe formula. The Mott–Bethe formula is given as follows:

$$\begin{array}{c} f^{e}(q)=\frac{m_{e}e^{2}}{8\pi h^{2}\varepsilon_{0}}\left(\frac{Z-f^{X}(q)}{s^{2}}\right),\\ =\frac{2\pi m_{e}e^{2}}{h^{2}\varepsilon_{0}}\left(\frac{Z-f^{X}(q)}{q^{2}}\right),\\ =\frac{2}{r_{H}}\left(\frac{Z-f^{X}(q)}{q^{2}}\right). \end{array}$$ (7)

Here, *h* is the Planck constant, and *r*_H_ is the in Bohr radius. Bohr radius is approximately 5.292 × 10^−11^ m and is given as follows:

$$r_{H}=\frac{h^{2}\varepsilon_{0}}{\pi m_{e}e^{2}}.$$ (8)

Despite its methodological simplicity, in which the form factor for the electrons is directly obtained from that of the x-ray, the Mott–Bethe formula yields remarkably accurate results for the *q* range (*q* > 1 Å^−1^) typically used for TR electron scattering experiments. The accuracy of the form factors calculated using the Mott–Bethe formula will be further discussed in Sec. IV A. Thanks to its advantageous accuracy, this formula has been extensively utilized in electron scattering studies for several decades. Meanwhile, the form factor for electron scattering shows more rapid changes with respect to *q* compared to that for x-ray scattering, because it is inversely proportional to the square of *q*. Therefore, the interference pattern of electron scattering, which indicates the cross term in the Debye equation [*I_M_* in Eq. (2)], exhibits sharp rise and decay. As mentioned earlier, these characteristics may explain the tendency to use the term “diffraction” even when describing electron scattering from non-crystal samples.

###### (2) Effect of acceleration voltage

The scattering intensity calculated using Eqs. (3) and (7) is independent of the acceleration voltage. Nonetheless, in reality, the scattering intensity of a relativistic electron with a high acceleration voltage differs from that of a non-relativistic electron. A comprehensive representation of the electron scattering intensity, encompassing scenarios involving relativistic electrons, can be expressed as follows:^25,72^

$$I(q)=\left(\sum_{i}f_{i}{\left(q\right)}^{2}+\sum_{i}\sum_{j\neq i}f_{i}(q)f_{j}(q)\frac{\;\text{sin}(qr_{ij})}{qr_{ij}}e^{-\frac{l_{ij}^{2}q^{2}}{2}}\right)\left(\frac{1-\alpha^{2}\;{\text{sin}}^{2}(\frac{2\theta }{2})}{1-\alpha^{2}}\right).$$ (9)

Here, 2*θ* is the scattering angle corresponding to *q*, *α* is defined using the speeds of the electron (*v_e_*) and light as follows:

$$\alpha =\frac{v_{e}}{c}.$$ (10)

In practice, the effect of the acceleration voltage can be considered as the scaling of the scattering amplitude, or form factor, by the square root of (1*−α*^2^sin^2^(2*θ*/2))/(1*−α*^2^). It should be noted that, in the case of a non-relativistic electron, *α* in Eq. (9) approaches 0, yielding Eq. (3).

###### (3) ELSEPA

Alternatively, ELSEPA, which is the acronym for Dirac partial-wave calculation of ELastic Scattering of Electrons and Positrons by Atoms, positive ions and molecules, can be employed to derive the form factors of atoms for electron scattering.^73,74^ Operating on the principles of the Dirac equation, it computes the scattering amplitude, or form factor, by considering the interaction between relativistic electrons and atoms. In comparison to the Mott–Bethe formula, ELSEPA is considered a theoretically advanced approach. The form factor of an atom obtained by ELSEPA contains element-specific phase information. Utilizing the form factors acquired through ELSEPA, one can calculate the scattering intensity of a molecule through an equation adapted from the conventional Debye equation as follows:

$$I(q)=\sum_{i}f_{i}^{e}{\left(q\right)}^{2}+\sum_{i}\sum_{j\neq i}f_{i}^{e}(q)f_{j}^{e}(q)\;\text{cos}(\eta_{i}(q)-\eta_{j}(q))\frac{\;\text{sin}(qr_{ij})}{qr_{ij}}e^{-\frac{l_{ij}^{2}q^{2}}{2}}.$$ (11)

Here, *η_i_*(*q*) indicates the phase of *i*th atom. The phase difference between two atoms is taken into account in the scattering intensity by a factor of cos(*η_i_*(*q*)−*η_j_*(*q*)). Another characteristic of the form factor obtained using ELSEPA is its inherent consideration of the effect of the acceleration voltage. Consequently, when utilizing the form factors obtained from ELSEPA, the scattering intensity computed using Eq. (11) remains accurate even for relativistic electrons.

#### 3. Anisotropic scattering intensity

In TR x-ray and electron scattering experiments, reactions are often initiated by photoexcitation via a pump laser pulse. Often the pump laser pulse is linearly polarized, and in this case, the excitation probability of a molecule is proportional to cos^2^*γ*, where *γ* is the angle between the light polarization and the transition dipole moment of the molecule. Since molecules exhibit varying excitation probabilities based on their orientation, the molecules in the excited state, as well as those remaining in the ground state, do not have random orientations after the photoexcitation. In such cases, the scattering signal detected on a 2D area detector due to photoexcited molecules can be described as a combination of isotropic and anisotropic scattering intensities. Specifically, the scattering intensity can be expressed using the following equation:^54–56^

$$\begin{array}{c} I(q,\theta_{q})\propto I_{0}(q)-P_{2}(\text{cos}\;\theta_{q})I_{2}(q)=I_{0}(q)-P_{2}(-\text{cos}\left(\frac{2\theta }{2}\right)\;\;\text{cos}\;\varphi )I_{2}(q). \end{array}$$ (12)

Here, cos*θ_q_* is defined as −cos(2*θ*/2)cos*φ*, where 2*θ* is the scattering angle, and *φ* represents the angle formed by the laser polarization and momentum transfer vector projected onto the detector [Fig. 1(b)]. *P*_2_ is the second-order Legendre polynomial, *I*_0_(*q*) is the isotropic scattering intensity, and *I*_2_(*q*) is the anisotropic scattering intensity. As shown in Eq. (12), *I*(*q*,*θ_q_*) is dependent on *φ* beside 2*θ*, confirming the anisotropic nature of the scattering intensity. *I*_0_(*q*) can be obtained through the Debye equation. *I*_2_(*q*) reflects the anisotropy arising from the nonrandom orientation of molecules and is calculated as follows.

$$I_{2}(q)=\sum_{i}\sum_{j\neq i}f_{i}(q)f_{j}(q)P_{2}(\text{cos}\;\beta_{ij})j_{2}(qr_{ij}).$$ (13)

In this equation, *β_ij_* is the angle between the transition dipole moment and *r_ij_*, and *j*_2_ is the second-order spherical Bessel function. It is worth noting that *I*_2_(*q*) varies with *β_ij_*, indicating its dependence on the direction of the transition dipole moment.

In practice, isotropic and anisotropic signals are extracted from a 2D scattering image and used for analysis. As depicted in Eq. (12), by plotting the scattering intensity of detector positions with the same *q* on the 2D scattering image against *P*_2_(cos*θ_q_*), −*I*_2_(*q*) and *I*_0_(*q*) can be derived as the slope and y-intercept, respectively. Repeating this procedure for all *q* values allows obtaining *I*_0_(*q*) and *I*_2_(*q*) for those *q* values. The acquired *I*_0_(*q*) and *I*_2_(*q*) offer information about the molecular structure and transition dipole moment.

### B. *Ab initio* calculation

IAM has the advantage of describing the scattering intensity of molecules with complex connectivity using a simple equation. In many cases, it has accurately depicted the scattering intensities of molecules. However, in recent studies, particularly those on gas-phase samples, inaccuracies in describing the scattering intensity using an IAM-based method have been highlighted.^75,76^ To address these inaccuracies, *ab initio* calculation, accounting for the complex electronic features of a molecule, can be employed in the calculation of the scattering intensity. In this approach, the wavefunctions of a molecule are determined through quantum calculations, and the scattering intensity is subsequently computed using information about the wavefunctions.^75,77^ Specifically, molecular structures, electronic states, and corresponding wavefunctions capable of describing the reaction of interest are obtained through quantum calculations. High-level simulations are often employed to generate a comprehensive trajectory representing the reaction of interest. Subsequently, theoretical scattering intensities corresponding to these molecular structures are computed through an *ab initio* approach that takes into account the derived wavefunctions. The assessment of the agreement between the calculated theoretical scattering intensities and the experimental data allows for the identification of the most suitable molecular structure or the validation of the simulation trajectory in describing the reaction. Unlike IAM, this method excels in accurately calculating the electron density or electrostatic potential of valence electrons, which are typically engaged in chemical bonds and ionization, resulting in theoretically more accurate scattering intensity. Nevertheless, a limitation arises as *ab initio* calculations pose increasing challenges with larger molecular sizes, primarily due to the time required for the *ab initio* calculations.

### C. Analyzing the difference scattering signal

In TR scattering experiments, the proportion of molecules in a sample undergoing the reaction initiated by the pump is generally limited. As a result, the scattering signal after the reaction initiation often closely resembles the scattering signal before the trigger. To elucidate changes in the scattering signal before and after the initiation of the reaction, the difference scattering signal [Δ*I*(*q*,*t*)], represented by the following equation, is utilized in the analysis of TR x-ray and electron scattering data.

$$\Delta I(q,t)=I(q,t)-I_{\text{ref}}(q).$$ (14)

Here, *I*(*q*,*t*) represents the scattering intensity at a time delay, *t*, with respect to the initiation of the reaction, and *I*_ref_(*q*) is the reference scattering intensity. Typically, the scattering signal obtained before the reaction trigger or a scattering signal acquired without any reaction trigger is used as *I*_ref_(*q*). The difference scattering signal encodes information about structural changes in molecules before and after the reaction. Utilizing the difference scattering signal for analysis has a couple of benefits: it (i) allows the depiction of subtle changes in the scattering signals arising from structural changes in the samples and (ii) offers the advantage of minimizing experimental artifacts, such as scattering from air.

## IV. COMPARISON OF TIME-RESOLVED X-RAY AND ELECTRON SCATTERING

X-rays and electrons share similar features in providing information about the structure of molecules through scattering. However, significant differences arise between x-ray and electron scattering in certain aspects such as scattering power and charge. These distinctions are briefly summarized in Table I. Such differences lead to significant variations in factors such as typical sample thickness and sample environment for the experiments. In this section, a detailed comparison of time-resolved scattering experiments using x-rays and electrons will be provided.

**TABLE I.** Comparison of key features between x-rays and electrons in the context of scattering. Notably, x-rays and electrons exhibit different charges and scattering powers, resulting in distinct experimental characteristics.

		x-ray (> 5 keV)	Electron (few MeV)	Electron (< 100 keV)
Origin of scattering		Interaction with electron	Interaction with electrostatic potential	
Charge (*e*)		0	−1	
Relative scattering power		1	10^9^	10^7^
Typical sample thickness	Solid	∼50 nm–10 mm	∼30–200 nm	∼20–200 nm
	Liquid	∼50–500 *μ*m	< 500 nm	None
	Gas	> 1 mm	∼100 *μ*m–4 mm	∼30 *μ*m–1 mm
Space charge effect		None	Relatively small	Relatively large
Relative sensitivity to hydrogen		Low	High	
Facilities for time-resolved scattering		Synchrotron, x-ray free-electron laser	MeV-UED (ultrafast electron diffraction) facility	keV-UED facility

### A. Scattering power

Typically, the scattering power of electrons is much larger than that of x-rays. According to an estimation by Pirenne, electrons have a scattering power approximately 10^6^ times stronger than x-rays.^21^ This estimation is derived from the equations for the raw scattering signal of x-rays [*I*^X^(*q*)] and electrons [*I^e^*(*q*)] scattered by an atom, which are given as follows.

$$I^{X}(q)=\frac{1+{\text{cos}}^{2}(2\theta )}{2}\frac{I_{0}}{R^{2}}r_{e}^{2}f^{X}{\left(q\right)}^{2},$$ (15)

$$I^{e}(q)=\frac{I_{0}}{R^{2}}f^{e}{\left(q\right)}^{2}=\frac{I_{0}}{R^{2}}\frac{4}{r_{H}^{2}}{\left(\frac{Z-f^{X}(q)}{q^{2}}\right)}^{2}.$$ (16)

Here, 2*θ* is the scattering angle corresponding to *q*, *I*_0_ is the intensity of the incident beam, and *R* is the distance from the measured position to the scattering sample. The terms *Z−f*^X^(*q*) in electron scattering and *f*^X^(*q*) in x-ray scattering have the same order of magnitude, and the term (1+cos^2^(2*θ*))/2 does not significantly affect the order of magnitude. Considering these, it may seem at first glance that the ratio of *I^e^*(*q*) to *I*^X^(*q*) can be compared simply using *r*_H_ and *r_e_*. However, since *I^e^*(*q*) and *I*^X^(*q*) are proportional to $r_{H}^{-2}$ and $r_{e}^{2}$, the ratio of *I^e^*(*q*) to *I*^X^(*q*) is proportional to $r_{H}^{-2}r_{e}^{-2}$, which is not unitless. To obtain a unitless ratio between the scattering intensities, a reference *q* value for comparison is necessary. Specifically, the ratio can be obtained as follows:

$$\begin{array}{c} \frac{I^{e}(q)}{I^{X}(q)}=\frac{\frac{I_{0}}{R^{2}}\frac{4}{r_{H}^{2}}{\left(\frac{Z-f^{X}(q)}{q^{2}}\right)}^{2}}{\frac{1+{\text{cos}}^{2}(2\theta )}{2}\frac{I_{0}}{R^{2}}r_{e}^{2}f^{X}{\left(q\right)}^{2}},\\ =\frac{{\left(\frac{4}{0.5292}\right)}^{2}\frac{1}{q^{4}}}{{\left(2.818\times 10^{-5}\right)}^{2}}\frac{{\left(Z-f^{X}(q)\right)}^{2}}{\frac{1+{\text{cos}}^{2}(2\theta )}{2}f^{X}{\left(q\right)}^{2}},\\ \approx \frac{10^{10}}{q^{4}}. \end{array}$$ (17)

Note that *r_e_* and *r*_H_ are in Angstrom units. This choice allows the use of *q* in inverse Angstrom units, typical for TR scattering experiments. Equation (17) shows that the relative scattering power of electrons and x-rays depends on the *q* value used for the comparison. If we compare the scattering power of electrons and x-rays for *q* at 10 Å^−1^, the well-known ratio of 10^6^ can be obtained.

Still, this estimation has a limitation that warrants consideration. The scattering intensity calculated using Eq. (16) does not account for the effect of the acceleration voltage. An electron scattering intensity that incorporates the effect of acceleration voltage can be described as follows:^25,72^

$$I^{e}(q)=\frac{I_{0}}{R^{2}}\frac{4}{r_{H}^{2}}{\left(\frac{Z-f^{X}(q)}{q^{2}}\right)}^{2}\left(\frac{1-\alpha^{2}\;{\text{sin}}^{2}(\frac{2\theta }{2})}{1-\alpha^{2}}\right).$$ (18)

Another aspect to consider when comparing the scattering power is the *q* value. In practice, a *q* range with a maximum *q* less than 10 Å^−1^ is commonly used in analysis rather than a *q* range extending to or exceeding 10 Å^−1^. The comparison of scattering power at 5 Å^−1^, using scattering intensities calculated by Eqs. (15) and (18), can be summarized as follows:

$$\begin{array}{c} \frac{I^{e}(q)}{I^{X}(q)}=\frac{{\left(\frac{4}{0.5292}\right)}^{2}\frac{1}{q^{4}}}{{\left(2.818\times 10^{-5}\right)}^{2}}\frac{{\left(Z-f^{X}(q)\right)}^{2}\left(\frac{1-\alpha^{2}\;{\text{sin}}^{2}(\frac{2\theta_{e}}{2})}{1-\alpha^{2}}\right)}{\frac{1+{\text{cos}}^{2}(2\theta_{X})}{2}f^{X}{\left(q\right)}^{2}}\\ \approx \frac{10^{10}}{5^{4}}\left(\frac{1-\alpha^{2}\;{\text{sin}}^{2}(\frac{2\theta_{e}}{2})}{1-\alpha^{2}}\right)\approx \frac{10^{10}}{5^{4}}\left(\frac{1}{1-\alpha^{2}}\right). \end{array}$$ (19)

Here, 2*θ_e_* are 2*θ*_X_ are the scattering angles corresponding to *q* for electrons and x-rays, respectively. Even for electrons with relatively low acceleration voltages used in keV-UED experiments, the scattering angle for the *q* region of interest remains small (less than 5°). Considering this, the factor proportional to the square of the electron scattering angle is disregarded in the last term of Eq. (19). In the case of non-relativistic electrons, the comparison suggests that the scattering power of electrons is approximately 10^7^ times stronger than x-rays. Furthermore, in MeV-UED experiments, where the acceleration voltage can reach 4 MeV, the *α* value is approximately 0.994. Taking this into account, at 5 Å^−1^ and an acceleration voltage of 4 MeV for electrons, the scattering power is approximately 10^9^ times stronger than x-rays.

**FIG. 2.**
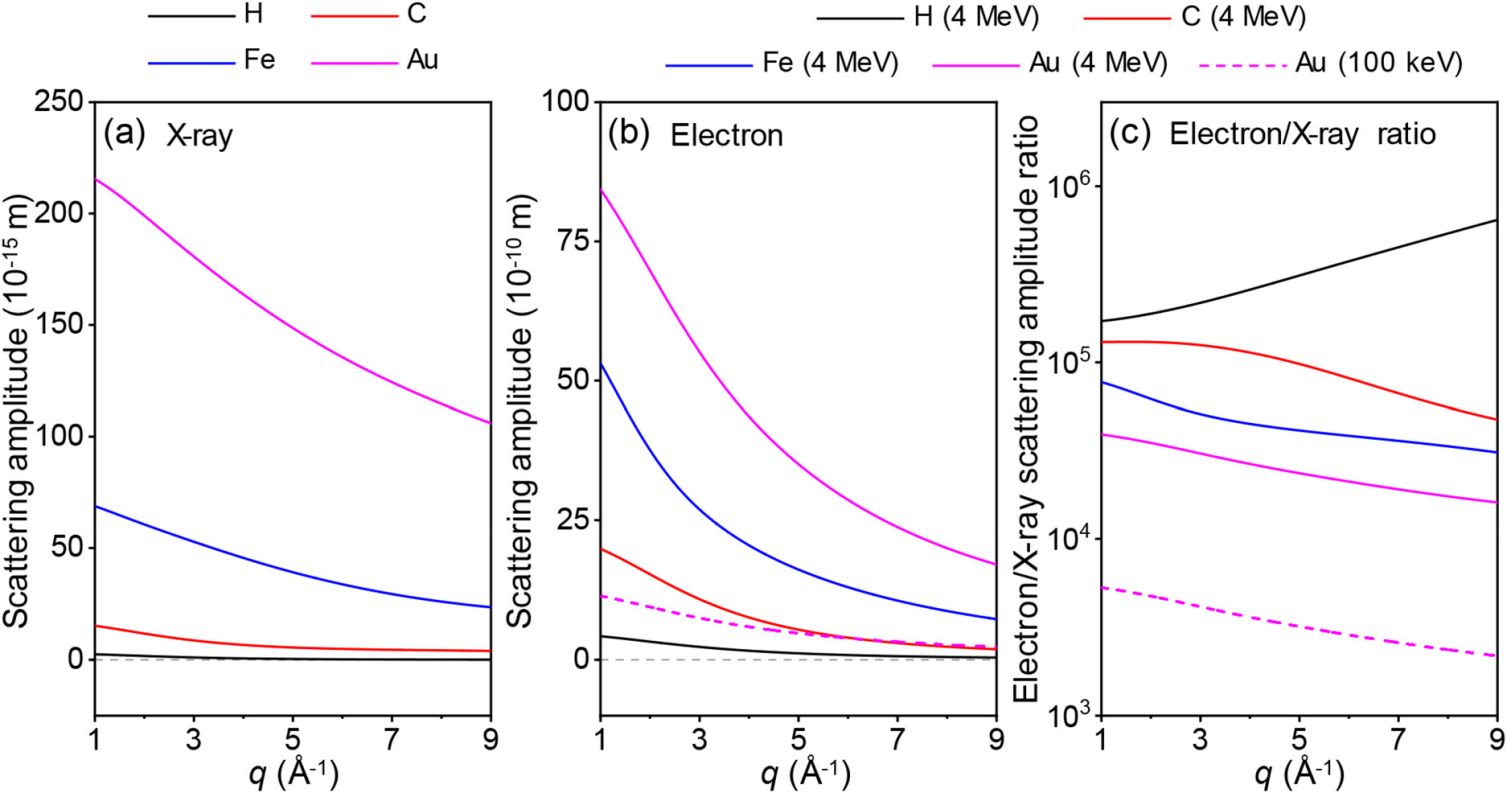
Scattering amplitudes of (a) x-rays and (b) electrons for H (black), C (red), Fe (blue), and Au (magenta). The scattering amplitudes were calculated based on atomic form factors. In the case of x-rays, the atomic form factors multiplied by the classical radius of the electron were considered equivalent to the scattering amplitudes. For electrons, the scattering amplitudes were determined by considering the dependence of scattering intensity on acceleration voltage. This involved multiplying the atomic form factors derived using the Mott–Bethe formula by a correction term for relativistic electrons. In (b), solid lines depict the scattering powers of electrons with an acceleration voltage of 4 MeV, while a dashed line represents the electron scattering amplitude for Au at 100 keV of acceleration voltage for comparison. Due to the difference in the speeds of electrons, the scattering amplitude of electrons with an acceleration voltage of 4 MeV is approximately 7.5 times larger than that of electrons with an acceleration voltage of 100 keV. (c) Ratios of the scattering amplitudes of electrons to those of x-rays for H (black), C (red), Fe (blue), and Au (magenta). The ratios for electrons with acceleration voltages of 4 MeV and 100 keV, are shown in solid and dashed lines, respectively.

Detailed scattering powers of atoms can be approximated as the squares of the scattering amplitudes for the atoms. In Fig. 2, the scattering amplitudes of x-rays and electrons are depicted for various atoms. While the atomic form factors for x-rays, derived from Eq. (4), are unitless, those for electrons obtained from Eq. (7) have a unit of length. To ensure consistency, the scattering amplitudes of x-rays were calculated by multiplying the x-ray form factors with the classical radius of the electron, thereby providing them with units of length. For electrons, the dependence of scattering intensity on the acceleration voltage was taken into account for the calculation of the scattering amplitudes. Specifically, a correction term, the square root of (1*−α*^2^sin^2^(2*θ*/2))/(1*−α*^2^), was applied to the atomic form factor of electrons determined by the Mott–Bethe formula, to obtain the electron scattering amplitudes. As illustrated in Figs. 2(a) and 2(b), the scattering amplitudes for x-rays and electrons with an acceleration voltage of 4 MeV show a substantial difference of approximately four to five orders of magnitude, corresponding to an approximately 10^9^ times difference in scattering powers. Another characteristic inferred from the comparison is that the ratio between the scattering amplitudes of electrons and x-rays increases as the atoms become smaller [Fig. 2(c)]. This suggests that electrons exhibit greater sensitivity to lighter atoms compared to x-rays. This characteristic will be discussed in detail in Sec. IV G. The difference in scattering power between the two scattering particles results in distinct penetration depths, indicating the extent to which scattering particles can penetrate through a material. If the thickness of the material is greater than the penetration depth, most of the scattering particles cannot pass through the material, and therefore, a scattering signal cannot be obtained using the transmission mode, as in an experimental setup shown in Fig. 1. In such a case, a different experimental setup, which measures the scattering signals reflected from the sample instead of those passing through the sample, can be used. The penetration depth is influenced by various parameters, such as the density of the material and the scattering power of the scattering particles. Because the scattering power of electrons is much greater than that of x-rays, the penetration depth for electrons is much shorter than that for x-rays in the same material.

**FIG. 3.**
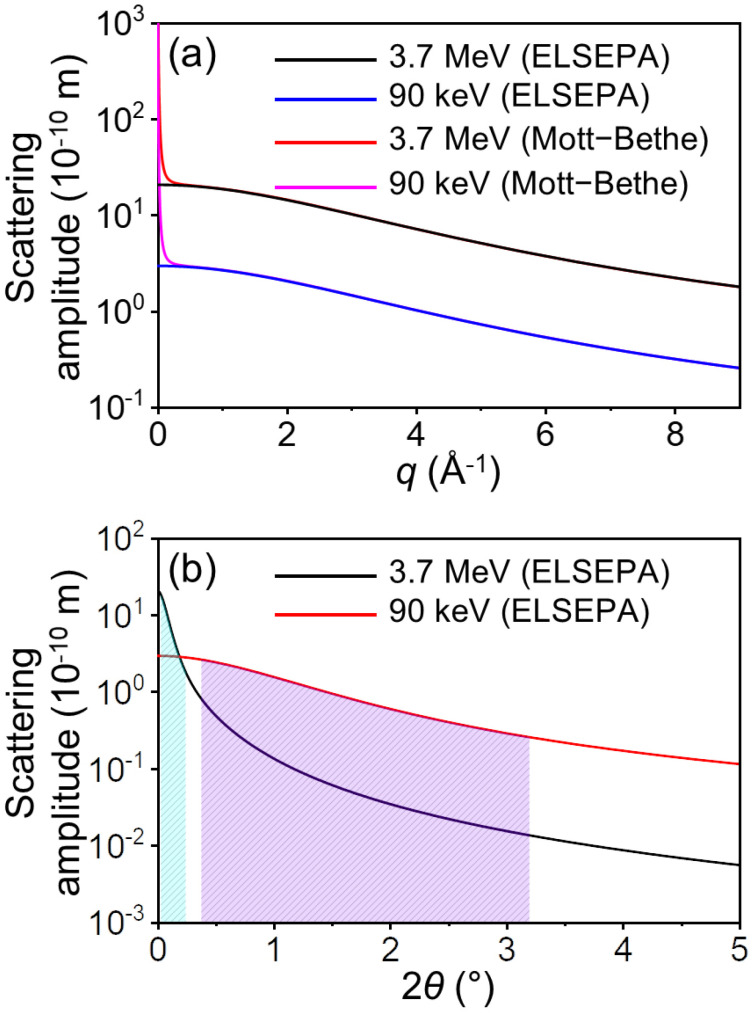
(a) Comparison of scattering amplitudes between relativistic electrons (3.7 MeV) and non-relativistic electrons (90 keV), calculated using ELSEPA and the Mott–Bethe formula. Scattering amplitudes for relativistic and non-relativistic electrons, obtained with ELSEPA, are shown in black and blue, respectively, while those estimated using the Mott–Bethe formula are depicted in red and magenta, respectively. (b) Scattering amplitudes of relativistic electrons (3.7 MeV, black) and non-relativistic electrons (90 keV, red) calculated using ELSEPA as a function of the scattering angle (2*θ*) instead of *q*. The light blue shading indicates the 2*θ* range for relativistic electrons, while the magenta shading represents the 2*θ* range for non-relativistic electrons within the *q* range of 1–9 Å^−1^.

Meanwhile, it is noteworthy that the scattering amplitude of relativistic electrons (4 MeV) surpasses that of non-relativistic electrons (100 keV), as shown in Fig. 2(b). The fact that the scattering power of relativistic electrons is stronger than that of non-relativistic electrons contradicts widely accepted knowledge. Taking this into consideration, we compared the scattering amplitudes of carbon for relativistic electrons (3.7 MeV) and non-relativistic electrons (90 keV) calculated using ELSEPA, an alternative method for calculating the form factors for electron scattering. The results from ELSEPA confirmed that the scattering amplitude of relativistic electrons is greater than that of non-relativistic electrons [Fig. 3(a)]. The previously established notion that non-relativistic electrons have a larger scattering amplitude than relativistic electrons is based on the comparison made at the same 2*θ*. When comparing at the same 2*θ*, the scattering amplitude of non-relativistic electrons is typically larger in accordance with the well-accepted notion [Fig. 3(b)]. Still, TR electron scattering data are typically described as a function of *q* rather than 2*θ* because 2*θ* can vary depending on the wavelength of the scattering particles. Considering this, we suggest that comparing scattering power at the same *q* is more appropriate. Furthermore, in the *q* region commonly utilized for typical TR electron scattering experiments (1 < *q* < 9 Å^−1^), the scattering amplitudes obtained through ELSEPA and those derived from the Mott–Bethe formula show excellent agreement, as shown in Fig. 3(a). The distinction between the scattering amplitudes acquired through ELSEPA and the Mott–Bethe formula becomes evident solely in the low *q* region, where *q* < 1 Å^−1^.

### B. Sample thickness

Given the considerably weaker scattering power of x-rays compared to electrons, x-rays penetrate much deeper into materials, resulting in significantly longer penetration depths. Consequently, TR x-ray scattering experiments can be conducted on thicker samples compared to TR electron scattering experiments. In TR x-ray scattering experiments performed at state-of-the-art facilities, samples of condensed matter (liquids and solids) composed of light atoms typically have a thickness of 10–100 *μ*m.^65,78–81^ In contrast, TR electron scattering experiments for condensed matter composed of light atoms are conducted with samples having a thickness of less than 1 *μ*m, often around 100 nm.^82,83^ Still, it should be noted that the thickness of the sample can vary significantly depending on factors such as the elements constituting the samples and phases of the samples. Solid samples typically contain heavy atoms more frequently and exhibit higher density than liquid samples, resulting in thinner thickness optimal for TR scattering experiments. For instance, a gold film with a thickness as small as 50 nm was employed even for a TR x-ray scattering experiment.^84^ For TR x-ray scattering experiments on gases, a millimeter-scale path length is widely used.^85,86^ Conversely, TR electron scattering experiments can use gas samples with a path length of several hundred *μ*m.^87,88^ The thinner sample thickness in electron scattering experiments can provide an advantage, typically requiring a smaller sample amount compared to x-ray scattering experiments and allowing for more efficient sample utilization. In recent TR x-ray and electron scattering experiments, studies are extending beyond prototypical samples to include those that are challenging to synthesize. Considering this aspect, the advantage of minimal sample consumption becomes significant. On the other hand, the requirement for a thinner sample in TR electron scattering can pose a limitation, as the preparation of thin samples is often more challenging than that of thicker ones.

### C. Sample environment

Due to the strong scattering power, the electron beam can be scattered out significantly even in the ambient pressure of air. Therefore, in TR electron scattering experiments, it is necessary to prepare vacuum conditions to obtain clear scattering signals from the target samples. In contrast, the x-ray, particularly hard x-ray with high energy commonly used in TR x-ray scattering experiments, offers an advantage of conducting experiments under ambient conditions. This makes the x-ray more versatile than electrons for performing TR scattering experiments under various conditions.

### D. Beam focusing

It is challenging to focus the x-ray using typical lenses because the change in refractive index depending on the materials is minute. Consequently, zone plates, mirrors, and compound lenses are often employed for x-ray focusing. The size of the focused x-ray pulse varies depending on the samples and experimental conditions. In recent TR x-ray scattering experiments using XFELs, the x-ray pulse can be focused to sizes below 100 *μ*m.^89–93^ Meanwhile, the charge of the electrons induces interesting properties in terms of focusing. On one hand, the charge of electrons makes it relatively easy to focus the electron pulse using electromagnetic force. Still, simultaneous repulsive forces among electrons within the pulse give rise to an obstacle to focus the electrons, which is known as the space charge effect. In MeV-UED, the space charge effect is significantly reduced compared to conventional keV-UED. In recent state-of-the-art MeV-UED experiments, electron pulses are typically focused to approximately 100–200 *μ*m.^94^

### E. Time and spatial resolutions

The time resolution of TR x-ray and electron scattering is influenced by various factors. Alongside elements such as the pulse duration of the pump pulse (typically a laser pulse), timing jitter between pump and probe pulses, and sample thickness, a critical determinant is the temporal width of the x-ray and electron pulses employed as probes. X-ray pulses generated at XFELs can have a pulse duration of less than tens of fs, resulting in time resolutions ranging from approximately tens of fs to 300 fs in TR x-ray scattering experiments at XFELs.^64,68,95–105^ In the case of electron scattering, the pulse duration of the electron pulse significantly depends on the beam current. Under the experimental conditions of state-of-the-art MeV-UED experiments, the pulse duration used for TR electron scattering can be reduced to less than 150 fs. Subsequently, TR electron scattering experiments conducted using MeV-UED facilities can achieve resolutions in the range of 100–300 fs.^32,49,51,60,82,83,106,107^ One method to enhance time resolution in TR x-ray scattering experiments involves the use of experimental setups such as timing tools. These apparatuses provide information about the actual time delay between the laser and x-ray pulses for each laser/x-ray pulse pair. By dramatically reducing the timing jitter between x-ray and laser pulses, overall temporal resolution can be improved. However, in the case of electron scattering experiments, systems similar to timing tools have not been employed, mainly because the timing jitters of TR electron scattering experiments are generally smaller than those of TR x-ray scattering experiments. The details regarding the pulse duration of XFELs and MeV-UED facilities are summarized in Table II.

**TABLE II.** Parameters for worldwide XFELs and MeV-UED facilities. Parameters to be considered for conducting TR x-ray and electron scattering experiments, such as the energy of scattering particles, the approximate number of particles per pulse, pulse duration, and repetition rate, are presented.

Facility	Scattering Particle energy (keV)	Number of particles/pulse	Pulse duration (FWHM) (fs)	Repetition rate (Hz)
XFELs				
LCLS	1–25	10^11^–10^13^	30	120
SACLA	4–20	10^11^	2–10	30–60
PAL-XFEL	2–15	10^11^–10^12^	25	60
European XFEL	5–20	10^12^	50	2.7 × 10^4^
SwissFEL	2–13	10^11^	1–50	100
LCLS-II-HE	4–20	10^10^–10^12^	30–100	120–10^6^
MeV-UED facilities				
SLAC	3.7 × 10^3^	1.3 × 10^4^	150	360
Osaka Univ.	2.5 × 10^3^	6.3 × 10^4^	60	10
BNL	3.0 × 10^3^	10^6^	100	48
KAERI	3.1 × 10^3^	3.8 × 10^6^	60	125
Shanghai Jiao Tong Univ.	3.0 × 10^3^	1.3 × 10^5^	30	100
LBNL	0.8 × 10^3^	1.6 × 10^4^	< 660	500

A crucial factor determining spatial resolution is the wavelength of scattered particles, linked to their energy. Spatial resolution is inversely proportional to the wavelength of a scattering particle, which, in turn, is determined by the energy of scattering particles. A higher energy of the scattering particles corresponds to a shorter wavelength, leading to improved spatial resolution in the experiment. The x-ray energy used in TR x-ray scattering is typically below 20 keV, corresponding to a wavelength greater than approximately 0.6 Å. In contrast, the electrons used for TR electron scattering experiments span energies from several tens of keV to several MeV. MeV-UED experiments have utilized electron energies up to approximately 4 MeV, corresponding to a de Broglie wavelength of roughly 0.003 Å. Consequently, under the identical experimental conditions except for the probe wavelength, electron scattering holds the potential to achieve superior spatial resolution compared to x-ray scattering due to the shorter wavelength. However, in practice, the spatial resolution of TR electron scattering experiments may not be significantly superior because only scattering data within a limited range of the scattering angle are typically utilized for the analysis.

### F. Number of observable Bragg peaks

Another critical aspect related to the wavelength of scattering particles is the number of observable Bragg peaks from a crystalline sample. The number of observable Bragg spots can be estimated using the Ewald sphere, an imaginary sphere in reciprocal space with a radius of 1/*λ* and passing through the origin. Bragg peaks from crystal samples can be represented as lattice points in reciprocal space, and those situated on or near the surface of the Ewald sphere are observable. In electron diffraction, where wavelengths of scattering particles are shorter, the surface of the Ewald sphere becomes more planar, allowing simultaneous observation of Bragg peaks on specific lattice planes in reciprocal space. Conversely, in x-ray diffraction, the surface of the Ewald sphere maintains a curved shape due to longer wavelengths, enabling the observation of Bragg peaks on various lattice planes. Consequently, x-ray diffraction typically yields a larger number of observable Bragg peaks. Because structure determination via crystallography requires data from Bragg peaks on various lattice planes, x-rays are more commonly employed in crystallography than electrons.

### G. Sensitivity to light atoms

**FIG. 4.**
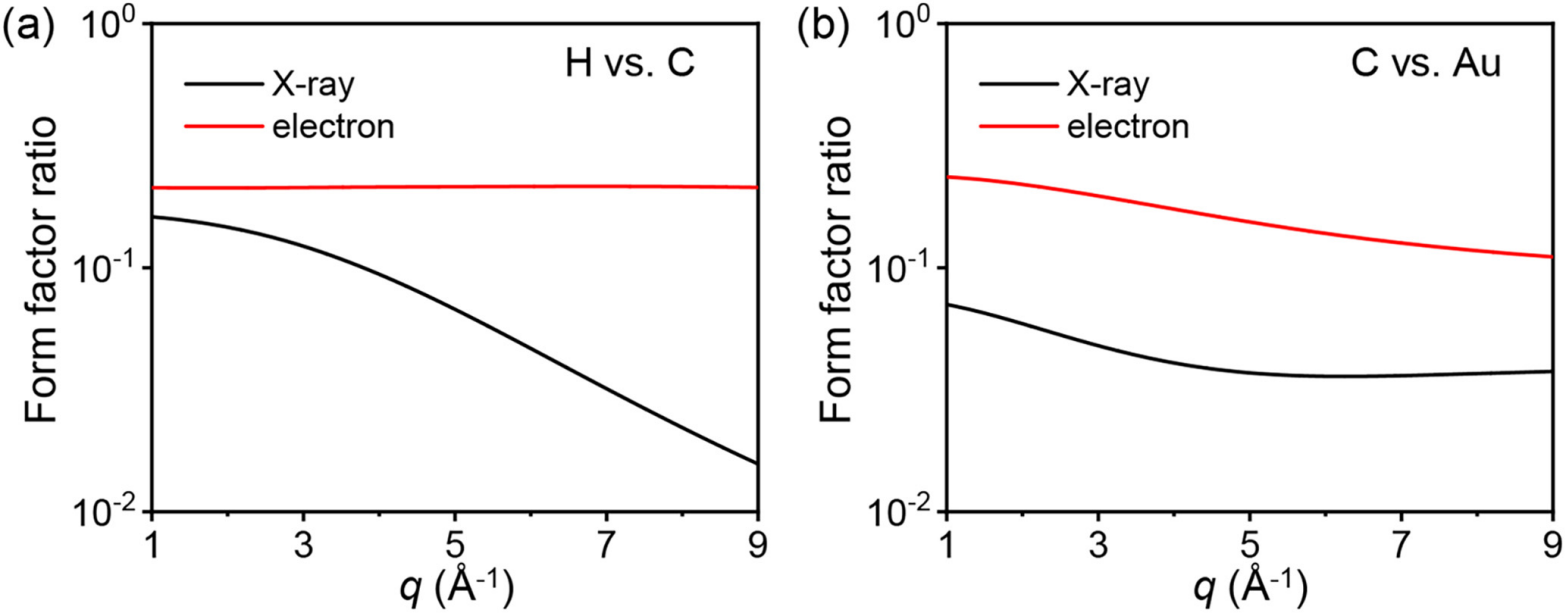
Ratios of the form factors for (a) hydrogen relative to carbon and (b) carbon relative to gold as a function of *q*. The ratios between the form factors of x-rays are shown in black, while those of electrons are shown in red. The form factors of electrons were calculated using the Mott–Bethe formula.

Electrons exhibit a higher sensitivity to light atoms than x-rays. As discussed in Sec. IV A, the relative scattering amplitude, which can represent the relative sensitivity, of electrons to x-rays increases as the atom becomes lighter. This suggests that, in comparison to electrons, the sensitivity of the x-ray scattering signal decreases more rapidly as the atom becomes lighter. Consequently, in a sample comprising various elements, the contribution of the lightest atoms to the overall scattering signal would be more prominent in electron scattering than in x-ray scattering. Similar results can be inferred when comparing the form factors of the same scattering particle for light and heavy atoms. For instance, the ratio of the form factor for hydrogen relative to carbon is larger for the electrons than for x-rays [Fig. 4(a)]. Likewise, the ratio of the form factor for carbon compared to that for gold is larger for the electrons [Fig. 4(b)]. The larger ratios indicate that the sensitivities to light atoms compared to heavier atoms are larger for electron scattering. Conversely, the heavier atoms have relatively enhanced sensitivities in x-ray scattering than in electron scattering. Additionally, for x-rays, the ratio of the form factor for hydrogen compared to that for carbon decreases significantly in the high *q* region [Fig. 4(a)]. On the other hand, for electrons, the ratio exhibits a minute dependence on *q*. Therefore, to effectively leverage the higher sensitivity of electron scattering to hydrogen atoms, securing the signal in the high *q* region is crucial.^25^

## V. X-RAY AND ELECTRON SOURCES FOR TIME-RESOLVED EXPERIMENTS

The recent establishment of XFELs and MeV-UED facilities marks a substantial leap forward in TR x-ray and electron scattering experiments. This section provides a brief overview of several XFELs and MeV-UED facilities. Each XFEL and MeV-UED facility exhibits unique characteristics, and some parameters essential for TR x-ray and electron scattering experiments, such as the energy of the scattering particle, are summarized in Table II.

### A. XFELs

#### 1. LCLS

Linac Coherent Light Source (LCLS), located in Menlo Park, the United States, is the world's first XFEL.^15^ As the inaugural XFEL, LCLS has facilitated the investigation of various dynamics, particularly those occurring in the ultrafast time domain, which were previously unattainable with traditional synchrotron-based TR x-ray scattering experiments. Furthermore, TR x-ray scattering experiments at the LCLS have explored various samples, encompassing solid, liquid, and gas phases. Presently, LCLS is in the process of upgrading to LCLS-II, as detailed later, with the anticipation of providing an even broader spectrum of research opportunities.

#### 2. SACLA

SACLA, an acronym for Spring-8 Angstrom Compact free electron LAser, is located in Sayo, Japan and is the second XFEL to begin operations worldwide.^16^ TR x-ray scattering experiments are predominantly conducted at the BL2 and BL3 beamlines, with a focus on samples in the condensed phase, especially those in the solid phase.

#### 3. PAL-XFEL

Pohang Accelerator Laboratory XFEL (PAL-XFEL) is located in Pohang, the Republic of Korea.^17^ The primary focus of TR x-ray scattering experiments at the PAL-XFEL is on samples in solid and liquid phases. Typically, the TR x-ray scattering experiments are performed at X-ray Scattering and Spectroscopy (XSS) and Nano Crystallography and coherent Imaging (NCI) instruments. Additionally, the PAL-XFEL has the capability to deliver tender x-rays and self-seeded beams with the highest brightness in the world,^108,109^ although these experimental conditions are not widely employed for TR x-ray scattering.

#### 4. European XFEL

The European XFEL, located in Schenefeld, Germany, generates x-ray pulses with a unique composition.^18^ Using a superconducting linear accelerator, it can generate an x-ray pulse train, which contains 2700 x-ray pulses with a pulse duration less than 100 fs, at a repetition rate of 10 Hz. The repetition rate between each pulse in a pulse train reaches approximately 4.5 MHz. Currently, there are ongoing efforts to effectively utilize this high repetition rate.^110–116^

#### 5. SwissFEL

The SwissFEL is located in Villigen, Switzerland.^19^ Currently, it provides the x-ray with relatively low energy (up to 12.4 keV) compared to other XFEL facilities. However, since this energy range is typically used for TR x-ray scattering experiments at other XFELs, it is expected that there would be no issues with performing TR x-ray scattering experiments at the SwissFEL. TR x-ray scattering experiments at the SwissFEL have predominantly focused on solid samples.

#### 6. LCLS-II

LCLS-II, represents an upgraded version of LCLS, with ongoing improvements.^117^ As the first XFEL utilizing continuous-wave superconducting accelerator technology, the LCLS-II anticipates a substantial increase in brilliance, potentially exceeding ∼1000-fold in the hard x-ray region (and ∼10000-fold in the soft x-ray regime) compared to the LCLS. A larger brilliance indicates the potential to produce the x-ray with increased flux, reduced divergence, or a combination of both. The long-term plan includes increasing the repetition rate from the current 120 Hz to 1 MHz. However, the upgrade to LCLS-II High Energy (LCLS-II-HE),^118^ which produces the hard x-rays commonly employed in TR x-ray scattering experiments, requires more time, leading to additional waiting periods for experimental execution. Nevertheless, such an upgrade for the hard x-ray holds the potential to broaden the scope of dynamic studies that were previously challenging with conventional XFELs. For instance, simulation results suggest that it would be feasible to observe vibrational motions in molecules consisting solely of light atoms, like ozone, using the TR x-ray scattering experiment at LCLS-II-HE, LCLS-II that provides high-energy x-rays.^119^

### B. MeV-UED facilities

#### 1. SLAC MeV-UED

Stanford Linear Accelerator Center (SLAC) MeV-UED, located near the LCLS-II, stands out as one of the most successful among many MeV-UED facilities developed to date, yielding ample high-impact research. Since the demonstration of the experimental setup in 2015,^20,94^ extensive research on numerous samples in solid and gas phases has been conducted using the SLAC MeV-UED facility. Recently, it has also pioneered TR electron scattering experiments on samples in liquid phase, marking a first case for UED facilities.

#### 2. KAERI MeV-UED

Korea Atomic Energy Research Institute (KAERI) MeV-UED, located in Daejeon, the Republic of Korea, has garnered significant recent attention for its achievement in producing a high-charge electron pulse of 0.6 pC. It boasts a very short pulse duration with a full width at half maximum (FWHM) of 60 fs, a minimal time jitter of less than 18 fs (FWHM), and a resultant temporal resolution of 73 fs (FWHM) with the use of an optical pump pulse of 45 fs (FWHM).^120,121^ This exceptional temporal resolution stems from the use of a pair of achromatic bends, also known as double-bend achromat (DBA), to correct the positive chirp of the electron pulse. This design allows higher-energy electrons to traverse longer paths, facilitating compression along the time axis within a single electron pulse, and ensuring constant arrival time to sample for each electron pulse, thereby minimizing time jitter.

#### 3. Other facilities

Beyond the mentioned MeV-UED facilities, various research institutions—such as Shanghai Jiao Tong University, Lawrence Berkeley National Laboratory, and Osaka University—are actively developing their own MeV-UED facilities. These endeavors go beyond the existing SLAC MeV-UED system, aiming to achieve higher temporal resolution by adopting DBA (Shanghai Jiao Tong University),^122^ enhancing repetition rates with slightly reduced acceleration voltages (HiRES from Lawrence Berkeley National Laboratory),^123^ or venturing into new domains like incorporating MeV-accelerated electrons into microscopy (Osaka University).^124^

### C. Comparison of XFELs and MeV-UED facilities

The most notable difference between XFELs and MeV-UED facilities lies in their size. In the case of the XFELs, devices to accelerate electrons to high energies (in the GeV regime) and undulators to generate x-rays typically require lengths on the order of kilometers. For example, the PAL-XFEL, LCLS, and SACLA have lengths of about 1.1, 3.5, and 0.75 km, respectivley. In contrast, the MeV-UED facilities have a relatively small size, making them suitable for lab-scale setups in universities or institutions. Moreover, the costs for setting up the MeV-UED facilities are comparatively much lower, fostering numerous initiatives to implement MeV-UED setups at institutional and university levels.

## VI. APPLICATION (PREVIOUS STUDIES)

**FIG. 5.**
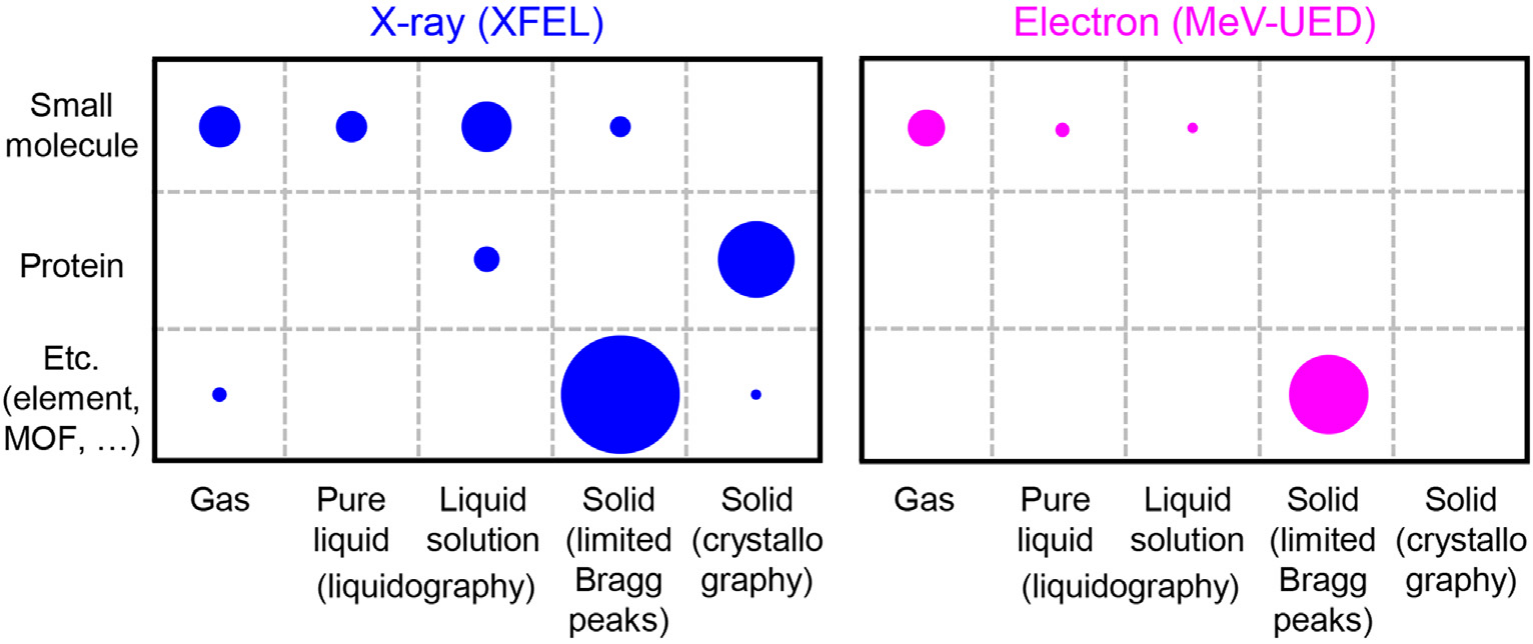
Scope and relative numbers of reported TR x-ray and electron scattering studies conducted at XFELs and MeV-UED facilities. These studies are categorized by the phases (columns) and types (rows) of the target samples. The size of each circle reflects the relative number of published studies within the respective category. TR x-ray scattering studies conducted at XFELs are illustrated in blue, whereas TR electron scattering studies performed at MeV-UED facilities are represented in magenta.

Since the development of XFELs and MeV-UED facilities, numerous TR x-ray and electron scattering studies have been reported. The scope and relative numbers of these TR x-ray and electron scattering studies vary depending on the phase and type of the target sample due to differences in facility numbers and the distinctive characteristics of x-rays and electrons, as illustrated in Fig. 5. This section will provide a concise overview of TR x-ray and electron scattering studies categorized by sample phase (solid, liquid, and gas).

### A. Solid (crystallography and limited Bragg peaks)

TR x-ray scattering studies on solid samples constitute the majority among all TR x-ray scattering studies (Fig. 5). While certain studies on single crystals relied on changes in the intensity of a select few Bragg peaks to deduce structural changes, the others achieved a direct visualization of the entire 3D electron densities via crystallography on single crystals, covering a significant portion of reciprocal space. In fact, XFELs, with their notably short pulse durations and large number of photons per pulse, spurred the emergence of serial femtosecond crystallography (SFX). In SFX, a single crystal encounters a single intense x-ray pulse before being discarded, and the diffraction signals obtained from a sufficient number of single crystals are merged to obtain a complete 3D electron density or crystal structure.^125–127^ Given the short pulse duration of x-ray pulses generated by XFELs, the diffraction by a crystal occurs before the x-ray induces damage to the crystal, allowing for obtaining an x-ray damage-free crystal structure through SFX. Time-resolved SFX (TR-SFX), which combines the pump–probe scheme and SFX, has been widely applied to investigate the structural changes in various single-crystal samples. Initially, TR-SFX studies have focused on protein samples,^96,99,104,128–154^ including myoglobin, photoactive yellow protein, and rhodopsin. These studies have delved into the structural dynamics of proteins, such as coherent protein motion and the ultrafast structural response of cofactors. Recently, the applications of TR-SFX have expanded to include a metal–organic framework (MOF), underscoring the versatility of TR-SFX.^155^

TR x-ray scattering experiments on different types of solid samples, such as polycrystalline samples and thin films of single crystals, have also proliferated. Typically, for these samples, structural changes are inferred by monitoring intensities of specific Bragg peaks. Experiments on such samples are frequently employed to observe photo-induced changes,^78,81,84,97,101–103,156–181^ such as phonon and phase transition. Some studies often investigated the structural changes induced by the pressure of laser shock.^182–200^

TR electron diffraction, simply called UED, experiments on solid samples have been actively conducted since the initial stages of MeV-UED development. In UED experiments on solid samples, structural changes are typically investigated using a limited number of Bragg peaks rather than employing crystallography. Via UED studies on solid samples,^32–51,201–207^ the structural changes in solids, such as phase transitions and lattice dynamics, have been scrutinized. One of the key phenomena studied using MeV-UED is the charge density wave (CDW), which is characterized by periodic modulation of electron distribution and lattice.^36,38–43^ In these studies, the investigation of CDW often involved analyzing changes in satellite peaks, distinctive features induced by the CDW, located in proximity to Bragg peaks.

### B. Liquid (liquid solution and pure liquid)

Similar to experiments with solid samples, TR x-ray scattering on liquid samples—also known as TR x-ray liquidography—has been extensively employed to investigate ultrafast dynamics that were challenging to observe in experiments using synchrotrons. This is achieved by using the x-ray with a short temporal width generated by XFELs. Representative examples of such studies involve investigating bond dissociation and association of molecules in liquid solution.^64,68,119,208–211^ These processes typically occur within a very short time frame, often on the order of picosecond (ps), posing difficulties to investigate via synchrotron-based experiments. The development of XFEL has made it possible to track these processes with time resolutions in the fs regime. This breakthrough allows for in-depth analyses, unveiling insights into the processes regarding the change in chemical bonds such as the speed of bond dissociation and providing a detailed understanding of reaction pathways when chemical bonds are formed. Meanwhile, the observation of molecular vibrations in the solution phase has become feasible as well due to improved time resolutions.^57,62,105,119,212–214^ For small molecules, the periods of vibrational motions are typically on the order of a few hundred fs, and the vibrational motions are damped within a few ps. Therefore, excellent time resolution is essential for detecting and studying molecular vibrations. Experiments utilizing the temporally short x-ray pulses generated by XFELs have met this condition and have been widely employed in the study of various molecular vibrations. In addition, various ultrafast processes, such as roaming-mediated isomerization, the rearrangement of the solvent cage induced by charge transfer, and solvation dynamics, have been investigated through TR x-ray scattering experiments on liquid solution samples.^64,98,215,216^ TR x-ray scattering experiments on liquid solution samples have been applied not only to small molecules but also to proteins such as the photosynthetic reaction center, myoglobin, and homodimeric hemoglobin.^100,217–221^ The solution phase, in comparison to crystals, provides an environment that is more similar to the physiological condition. Consequently, TR x-ray scattering experiments on protein solutions have provided insights into how structural changes of proteins occur in environments akin to physiological conditions. Additional details about TR x-ray scattering experiments on samples in the liquid phase are available in other review papers.^28,222,223^

TR x-ray scattering has been conducted on pure liquid samples as well, albeit less frequently than on liquid solutions.^65,69,79,80,224–226^ Representative studies on pure liquid samples include the investigation of the phase transitions and changes in molecular alignment induced by the optical Kerr effect.

Liquid-phase UED, commonly known as LUED, is a relatively recent experimental technique, and the number of studies conducted using LUED remains limited. The limited number of LUED studies, compared to TR electron scattering studies in other phases, is attributable to several technical challenges. First, the need for ultra-thin liquid sheets presents a significant challenge.^82,83,227,228^ As highlighted in Sec. IV A, the scattering power of electrons is much greater than that of x-rays, resulting in a significantly shorter penetration depth for electrons than for x-rays in the same material. This means that TR electron scattering requires the use of very thin (<1 *μ*m) liquid sheets in a high vacuum, whereas, in contrast, for TR x-ray scattering, much thicker (>100 *μ*m) liquid sheets can be used under ambient pressure conditions. Additionally, the requirement for a high-vacuum environment in LUED experiments presents an additional challenge. The creation of ultra-thin liquid sheets in a vacuum typically involves the use of microfluidic chip technology,^82,83,227,228^ wherein liquid is first flowed through a central channel to form a cylindrical jet. This jet is then compressed into a sheet form by streaming helium (He) gas at appropriate pressure through adjacent channels, pressing against the liquid jet from both sides. In this setup, the main factors that challenge the vacuum environment are the liquid and the He gas, with the liquid being the more dominant. Consequently, maintaining a high vacuum within the chamber requires effective capture of the injected liquid sheets, necessitating the use of specialized equipment such as liquid nitrogen traps or peristaltic pump systems that remove the captured liquid without freezing it and expel it outside the chamber. Notably, when employing liquid nitrogen traps, there is a need for periodic replacement of the reservoirs of the traps once they are filled with frozen liquid within the vacuum. These factors significantly contribute to the increased technical complexity associated with LUED studies. Nevertheless, nowadays, the generation of jets with thicknesses in the range of several hundred nanometers has become achievable, and experiments are typically performed using these thin jets.^227^ A representative sample investigated using LUED is neat water, with a focus on the changes in hydrogen bonding upon photoexcitation.^82,83^ Particularly, the development of MeV-UED has significantly increased time resolution, enabling the observation of the formation of water complexes in the ultrafast time domain. Additionally, LUED has been applied to study dynamics related to bond dissociation in triiodide anion.^228^

### C. Gas

Gas samples generally have a significantly lower number density of molecules compared to solid or liquid samples. Therefore, obtaining x-ray scattering signals with a sufficient signal-to-noise ratio from gas samples has been a highly challenging task, considering the low scattering power of the x-ray. However, with the development of XFEL, the number of photons per x-ray pulse has increased dramatically, enabling TR x-ray scattering experiments on gas-phase samples. Recently, TR scattering experiments on gas samples have been actively conducted using both electrons and x-rays.

TR x-ray and electron studies on gas samples often incorporate quantum calculations in the analysis to obtain the trajectories of molecules under reactions initiated by the pump. The isolated nature of the molecules eliminates the need to account for interactions with neighboring molecules, simplifying the quantum calculations. Moreover, when the target molecule consists of a small number of relatively light atoms, which is typical for gas samples, the computational cost remains relatively low. Using these advantages, high-level simulations such as *ab initio* multiple spawning (AIMS) simulations and surface hopping are frequently used for the analysis of gas-phase TR x-ray and electron scattering data.^229–232^ Although less common, even scattering intensities are occasionally calculated using *ab initio* methods^75,76^.

TR x-ray scattering has been employed to study the ultrafast structural dynamics of gas-phase samples,^58,61,63,66,67,95,233–243^ such as vibrations and bond dissociation. In particular, as gas molecules have no interaction among them, the x-ray scattering signal from the gas sample contains information solely about the electron density within the molecule. This characteristic enables TR x-ray scattering experiments on gas samples to effectively capture subtle changes within the molecule. Leveraging these advantages, some studies have assigned the detailed nature of the excited states upon photoexcitation based on the obtained scattering signal.^61,63,66^

Given the strong scattering power of electrons, UED is highly effective for materials with low density, as in the case of gases. Indeed, since the development of MeV-UED, numerous MeV-UED experiments have been conducted on gas samples,^59,60,75,76,106,107,244–247^ particularly on the gases composed of light atoms. A notable application of gas-phase UED is the investigation of pericyclic reactions, revealing conformer-specific dynamics and orbital rehybridization occurring during the reactions.^106,245,247^ Furthermore, a recent gas-phase UED experiment imaged the birth of ions and the associated structural dynamics in isolated conditions.^107^

### D. Comparison of XFELs and MeV-UED facilities for time-resolved scattering

A crucial parameter for comparing different XFELs and MeV-UED facilities lies in the scattering intensity they can achieve within the same time frame, related to the attainable signal-to-noise ratio for a given duration. In recent TR x-ray and electron scattering experiments, samples for the experiments have progressed from traditional simple model systems to challenging-to-synthesize systems. Performing experiments on such systems incurs increasing costs, presenting a practical limitation. By achieving a higher scattering intensity within a given time frame, it is possible to minimize the required sample volume through the reduction of sample jet thickness and data collection time, consequently alleviating sample preparation costs.

To estimate the expected scattering intensity or scattering events per unit time, parameters related to the experimental facilities—such as the relative scattering power of the scattering particles, the number of scattering particles per probe pulse, and the repetition rate of the probe pulses—should be considered. Additionally, parameters representing samples, including concentration and thickness, need to be taken into account. The expected signal-to-noise ratios can be roughly estimated as the square root of the expected scattering events per unit time. Table III provides expected signal-to-noise ratios and scattering events per second for TR scattering experiments on gas and liquid solution samples performed at typical XFEL facilities, keV-UED facilities, and MeV-UED facilities. It should be noted that these estimations are based on representative values for the mentioned parameters, and actual values may vary depending on the precise parameters regarding experimental facilities and samples.

**TABLE III.** Comparison of the relative signal-to-noise ratios and relative numbers of scattering events per second for TR x-ray and electron scattering experiments. The values for the experiment on gas and liquid solution at a typical XFEL facility, upgraded XFEL facility, keV-UED facility, MeV-UED facility, and upgraded MeV-UED facility are provided. The accompanying parameters used to calculate the relative signal-to-noise ratios and relative numbers of scattering events are presented as well. Parameters related to experimental facilities are approximated based on the specifications of representative facilities for XFEL (LCLS), upgraded XFEL (LCLS-II-HE), keV-UED facility (M. Centurion's group in University of Nebraska-Lincoln), MeV-UED facility (SLAC MeV-UED), and upgraded MeV-UED facility (KAERI MeV-UED-II). Those related to samples were selected from typical values used in TR scattering experiments. The relative number of scattering events was estimated as a product of the relative scattering power of the scattering particles, the number of scattering particles per pulse, the repetition rate, the relative sample concentration, and the relative sample thickness. The relative signal-to-noise ratio was estimated as the square root of the relative number of scattering events per second. The parameters are not absolute but vary depending on the specific experiment facility and experimental conditions employed. For instance, the number of scattering particles per pulse of an XFEL is dependent on the energy of the x-ray used in the experiment.

			XFEL		Upgraded XFEL		keV-UED		MeV-UED^a^		Upgraded MeV-UED^a^		
Relative scattering power of scattering particles			1		1		10^7^		10^9^		10^9^		
Number of scattering particles/pulse			1.0 × 10^12^		1.0 × 10^12^		1.0 × 10^4^		1.3 × 10^4^		1.9 × 10^7^		
Repetition rate (Hz)			120		3.0 × 10^5^		1.0 × 10^3^		360		250		
Relative number of scattering events per second (/10^14^)			1.2		3.0 × 10^3^		1		47		4.8 × 10^4^		
Concentration (mM) for			0.5	10	0.5	10	0.5	10	0.5	10	0.5	10
Gas		Liquid solution										
Sample thickness (*μ*m) for			2000	50	2000	50	2000	0.2	2000	0.2	2000	0.2
Gas		Liquid solution										
Relative^b^ number of scattering events per second for			2.0	1	5000	2500	1.7	3.3 × 10^−3^	78	0.16	7.9 × 10^4^	160
Gas	Liquid solution											
Relative^c^ signal-to-noise ratio^d^ for			1.4	1	71	50	1.3	0.058	8.8	0.40	280	13
Gas		Liquid solution										

^a^ Here, the efficiency of the detecting system is not considered for the estimation of the relative number of scattering events per second and signal-to-noise ratio. In reality, the efficiency of the detecting system is lower for MeV-UED experiments compared to keV-UED or x-ray scattering experiments because the direct bombardment mode, which is routinely utilized for keV-UED and x-ray scattering experiments, could not be easily used for MeV-UED experiments. Therefore, the relative number of scattering events per second and signal-to-noise ratio for current and upgraded MeV-UED facilities presented here are overestimated.

^b^ The values in this row are scaled down by a factor of 6.0 × 10^16^, which corresponds to the value used for x-ray scattering experiments on liquid solution samples at a typical XFEL.

^c^ The values in this row are scaled down by a factor of 2.4 × 10^8^, which corresponds to the value used for x-ray scattering experiments on liquid solution samples at a typical XFEL.

^d^ We assume that the relative signal-to-noise ratio is simply proportional to the square root of the relative number of scattering events per second, assuming the noise level is constant.

## VII. OUTLOOK AND CONCLUSION

In the last two decades, TR x-ray and electron scattering has undergone significant advancements, especially with the emergence of XFELs and MeV-UED facilities. Nonetheless, there remain opportunities for further development in TR x-ray and electron scattering. Recently, several XFELs and MeV-UED facilities such as LCLS and KAERI-UED are undergoing or planning upgrades to enhance the capabilities of providing x-ray and electron pulses with a larger number of scattering particles per pulse or a higher repetition rate than before. For instance, with the ongoing upgrade of XFEL facilities, the relative number of scattering events per unit time is expected to increase by approximately 1000-fold. These upgrades are expected to contribute to the reduction of the amount of sample and time required for experiments. The expected relative signal-to-noise ratios and relative number of scattering events per unit time, based on the anticipated specifications after the upgrades, are listed in Table III. Another experimental advancement can involve a scheme that utilizes x-rays and electrons together. When used as the probes in TR scattering experiments, x-rays and electrons perform similar roles, providing information about the molecular structure. However, when utilized as the pump, they can function differently. For instance, x-ray photons can excite core electrons, while electrons can induce molecular ionization. Considering the distinct roles of x-rays and electrons as pumps, it is possible to conduct TR scattering experiments using schemes such as x-ray pump–electron probe or electron pump–x-ray probe. Additionally, experiments employing schemes like x-ray pump–x-ray probe or electron pump–electron probe are also viable. In fact, x-ray pump–x-ray probe experiments are already feasible at some XFELs such as LCLS and SACLA.^139,143,170,248–250^

There is potential for advancements not only in the experimental aspects but also in the methods employed for analyzing the scattering signal. Currently, *ab initio* calculations for scattering intensity primarily focus on gas samples composed of light atoms. Anticipating advancements in computing power and calculation methods, this approach is expected to extend to samples containing multiple heavy atoms or samples in other phases. Concurrently, the rapidly advancing field of deep learning holds promise for the analysis of TR scattering data. Scattering intensity from a sample is typically measured in the form of 2D images, and deep learning has shown significant strength in the processing of such images. Consequently, leveraging deep learning for the analysis of 2D scattering images is anticipated to directly extract more information, including anisotropy, compared to the conventional reduction and analysis of data into 1D scattering curves.

In summary, although x-rays and electrons exhibit different characteristics in terms of scattering power and charge, both can be used to unveil molecular structures. Based on this common ground, TR scattering using x-rays and electrons has become a prominent experimental tool for studying the structural dynamics of molecules. Ongoing construction and upgrades of XFELs and MeV-UED facilities are expected to provide extensive opportunities for experiments and expand the range of samples available as targets for TR x-ray and electron scattering. In addition, the distinct characteristics of x-rays and electrons offer researchers the chance to select and conduct TR x-ray or electron scattering experiments tailored to their specific objectives. These advantages and advancements in TR x-ray and electron scattering are expected to foster a broader community of scientists utilizing this technique to investigate the structural dynamics of molecules.

## Data Availability

The data that support the findings of this study are available from the corresponding author upon reasonable request.
